# Wastewater Surveillance for Group A *Streptococcus pyogenes* in a Small City

**DOI:** 10.3390/pathogens14070658

**Published:** 2025-07-03

**Authors:** Olivia N. Birch, Frankie M. Garza, Justin C. Greaves

**Affiliations:** Department of Environmental and Occupational Health, School of Public Health, Indiana University-Bloomington, 2719 E 10th Street, Innovation Center, Bloomington, IN 47408, USA; onbirch@iu.edu (O.N.B.); francescagarza57@gmail.com (F.M.G.)

**Keywords:** wastewater-based epidemiology, GAS pharyngitis (strep-throat), viral respiratory pathogens, filtration, *Streptococcus pyogenes*

## Abstract

*Streptococcus pyogenes* is a bacterial pathogen known to be the causative agent in many different illnesses, with Group A Streptococcus (GAS) pharyngitis (strep throat), being one of the more prevalent. The spread and severity of GAS pharyngitis can grow exponentially if individuals are not taking the proper precautions. Wastewater surveillance has been used to test for numerous different pathogens that humans spread throughout a community and in this study, we utilized wastewater surveillance to monitor GAS pharyngitis in a small city. Over a year, 57 wastewater influent samples were tested for *S. pyogenes* and three commonly tested respiratory viruses (Respiratory Syncytial Virus (RSV), SARS-CoV-2, Influenza A). Three microbial indicators and population normalizers (CrAssphage, Pepper mild mottle virus (PMMoV), and Mycobacterium) were tested as well to compare and contrast each indicator’s value and range over time. Wastewater data was then compared to publicly available search term data as clinical data was not readily available. There was a high correlation between the collected molecular data and the publicly available search term data for *Streptococcus pyogenes*. Additionally, this study provided more information about the seasonal trend of *S. pyogenes* throughout the year through molecular data and allowed for the ability to track peak infection months in this small city. Overall, these results highlight the substantial benefits of using wastewater surveillance for the monitoring of GAS pharyngitis. This study also provides helpful insights into future studies about the prevalence of respiratory bacteria and their seasonal trends in wastewater, allowing for public health systems to provide mitigation strategies.

## 1. Introduction

Group A Streptococcus (GAS), *Streptococcus pyogenes*, is a human-specific pathogen known to cause both non-invasive and invasive diseases [1,2]. This bacterium has led to serious GAS infections in 18.1 million people, resulting in approximately 500,000 deaths annually [2]. *S. pyogenes* can cause a range of infections, from mild conditions such as Group A Streptococcal (GAS) pharyngitis (strep throat) and Streptococcal pyoderma, to severe invasive diseases like bacteremia (septicemia), Streptococcal toxic shock-like syndrome (STSS), and necrotizing soft tissue infections, including necrotizing fasciitis [3]. At the progression of the 20th century, there was a high surge and prevalence of this bacterium, but more recently, cases have significantly reduced. However, recorded severe cases have still been observed in multiple different countries around the world [4]. GAS pharyngitis (strep throat) is currently the most prevalent infection caused by *S. pyogenes* [1]. This acute upper respiratory infection mainly affects children of school age but is able to impact adults as well, with a sudden onset of fever, sore throat, and pain in the throughout the respiratory tract [1,3]. *S. pyogenes* is primarily passed along to other individuals through respiratory droplets by direct or indirect contact [5]. *S. pyogenes* is extremely contagious in all of its disease forms and can be a large threat to numerous populations worldwide [2,4].

The traditional epidemiological surveillance method for respiratory pathogens involves patients willingly admitting themselves to medical facilities to be tested and typically only occurs after the onset of symptoms [6,7,8,9]. Wastewater-based epidemiology is an emerging tool as it is not reliant on patient medical admissions and an entire community can be monitored at the same time using concentrations of pathogens in sewage [7,8]. Amplified by the coronavirus disease 2019 (COVID-19) pandemic, wastewater surveillance was utilized alongside patient admissions and testing to provide a more comprehensive view of the spread and prevalence of COVID-19. The addition of wastewater surveillance allowed for the incorporation of asymptomatic infections as well as those not initially tested, which can serve as an early warning tool for spreading illnesses [3]. Lastly, the use of wastewater testing for pathogens provides the ability for a holistic approach to pathogen prevalence and monitoring in order to encompass an entire population, which will include some infections that go untested or are missed during medical appointments [6,10]. These benefits make wastewater surveillance distinctly suitable and beneficial for monitoring *S. pyogenes* in communities that struggle with maintaining outbreaks as there has been an increase in GAS infections post pandemic [3]. Recently, there has only been one study carried out by Shrestha et al. in 2024 observing the prevalence of *S. pyogenes* in wastewater [3]. This study observed multiple detections of this pathogen in wastewater but was limited to the local area in Kofu City, Japan. Our study hopes to add crucial information alongside this study to help provide useful insights into the prevalence of *S. pyogenes* in wastewater and provide the potential for an early warning system.

In this study, we collected wastewater data on *S. pyogenes* over the span of one year and compared it to search data from Google Trends. In the modern age, individuals are forgoing making medical appointments about their symptoms and instead are turning to the internet. Due to the vast amount of public information on the internet, a search of a common illness can allow an individual to view the common symptoms, the infectivity period, and the appropriate time to see a medical professional based on the severity of symptoms [3,11]. Any searches on Google are compiled onto an accessible site that allows researchers to see the prevalence of searches in any given area during a given period. Using this data allows for data trends to be viewed and compared over time [3,11,12]. Our study sought to compare various proposed microbial indicators (mycobacterium, crAssphage, and pepper mild mottle virus (PMMOV)) to determine which is best suited for population normalization and quantification. Lastly, our study compared *S. pyogenes* values with the concentrations of other respiratory pathogens of recent importance (influenza A virus, Respiratory Syncytial virus, and SARS-CoV-2) to determine how the levels of each pathogen differ. Ultimately, the data from this study highlighted the importance of WBE in monitoring respiratory bacteria, like *S. pyogenes*, in communities where the disease is endemic in.

## 2. Materials and Methodology

### 2.1. Sample Collection and Site Selection

In total, 57 primary influent sewage samples, post grit screening, were collected over the course of a year from the main Wastewater Treatment Plant (WWTP) in Bloomington, Indiana. Bloomington is characterized as a small city with a population of just over 75,000 [13,14,15]. The main WWTP serves approximately 80% of the residents living within city limits and can treat up to 15 million gallons of wastewater per day. The samples were collected from 1 February 2023 to 28 February 2024, on Wednesday every week (2 samples per week). Samples were collected as 24 h composite samples in sterile polypropylene (PP) 50 mL tubes, transported in a cooler and frozen (−20 °C) within 2 h of collection, and samples remained stored at −20 °C until processing.

### 2.2. Sample Concentration and DNA/RNA Extraction

50 mL of Wastewater samples were acidified to a pH of 3.5 then 500 µL of a known concentration of bovine coronavirus solution [5.42–5.94 log_10_ gene copies/L] was added to the sample as a control. Filtration was then performed through an electronegative 47 mm diameter and 0.45 µm pore size mixed cellulose ester filter (Cat. No. HAWG047S6, Millipore Sigma, Burlington, MA, USA) based on previous studies [16,17]. Filters were transferred to 2.0 mL PowerBead tubes (Cat. No. 1103225, Qiagen, Germantown, MD, USA) and stored at −20 °C for DNA/RNA extraction. The Allprep PowerViral DNA/RNA extraction kit (Cat. No. 28000-50, Qiagen, Germantown, MD, USA) was used for the extraction of both DNA and RNA according to the manufacturer instructions.

### 2.3. Microbial Quantification by dPCR

The microbial quantification was performed using the QIAcuity™ Four Platform System (Cat. No 911042, Qiagen, Germantown, MD, USA), the QIAcuity Nanoplate 26k (Cat. No 250001, Qiagen, Germantown, MD, USA), and the QIAcuity™ Probe PCR Kit (Cat. No. 250132, Qiagen, Germantown, MD, USA). The PCR mix contained 1X Qiacuity OneStep Advanced Probe Master Mix, 0.8 μM of each primer and 0.4 μM of each probe, and 5 μL of target DNA for a volume of 40 μL. For RNA viruses, such as PMMOV, RSV, H1N1, and SARS-CoV-2, reverse transcriptase was performed using Qiacuity OneStep Advanced RT Mix at 50 °C for 65 min. The primers used for the quantification of PMMoV, RSV, SARS-CoV-2, H1N1, non-tuberculosis mycobacterium, and Group A *S. pyogenes* are described in Table S1. The primers and probes were synthesized by IDT. The probes were double quenched by the Zen™ internal quencher (IDTDNA.com) and the 3′ Black Hole™ quencher. Using DNA controls, the lowest detection limit was estimated for all PCR assays using a dilution series and was calculated to be 1.4 gene copies/PCR reaction for all targets, which represents 400 gene copies (gc)/L (2.6 log_10_ gene copies/L). Bovine coronavirus was used as an extraction control, resulting in 10–15% efficiency. Samples that resulted in non-detection were included in the results as the limit of detection. The molecular fecal indicators crAssphage and PMMoV were quantified using dPCR techniques as described above, and with the primers and probes previously published [18,19]. Primer and probe set selection were determined based on published, peer-reviewed assays conducted using NCBIBLAST [16,20,21,22,23,24,25].

### 2.4. Particle Association Experiment

Additional primary influent wastewater samples were collected from the Dillman WWTP between September and December 2023. Samples were then filtered through a cascade filtration format as described previously by da Silva et al. [26]. Briefly, 50 mL of each sample was sequentially filtered through 5 μm, 0.45 μm, and 0.025 μm mixed cellulose ester filters. Sample volumes were optimized prior to experimentation to maintain constant filter flow rates and minimize plugging. Filter sorption of ‘free’ microorganisms or particles was not directly assessed, i.e., particle sizes were operationally defined by collection on the appropriate filter. Flow rates were also not directly maintained or measured, but the volumes used (50 mL) were previously tested on wastewater samples to ensure efficient filtration and concentration of fecal targets with no filter clogging. The three filters were stored at −80 °C in bead tubes for DNA/RNA extractions and dPCR quantification as mentioned previously [13,16,18].

### 2.5. Data Analysis

Concentration of viruses (gc/L) was calculated using the following formula:

$$\frac{gc}{L}=\frac{gc}{uL}\times \frac{Vol\;PCR\;reaction\;(40\;uL)}{Vol.\;NA\;analyzed\;(10\;uL)}\times \frac{Vol.\;NA\;eluted\;(100\;uL)}{vol\;sample\;(0.05\;L)}.$$

Analysis of normality and Analysis of Variance analyses (ANOVA) were performed using GraphPad Prism v 10 (Boston, MA, USA), where factors such as (independent variables) months, filter type, and microbial type were considered. Tukey pairwise comparisons were performed as post hoc analysis after ANOVA. The significant difference between pathogens are indicated in the figures by connecting bars where the one asterisk (*) indicates a *p* value < 0.05, two asterisks indicate *p* < 0.01, three asterisks indicate *p* < 0.001, and four asterisks indicate *p* < 0.0001 To determine the correlation between the concentration of both viruses in the samples, Spearmen’s correlation analysis and graphics were made using GraphPad Prism v 10 (Boston, MA, USA).

### 2.6. Comparison with Google Trends Data

As individual clinical surveillance of *S. pyogenes* is not performed within this region, we decided to compare our results with search term data through the use of Google Trends. Google Trends tracks the frequency of searches for a term in a specific region, comparing it to the total search volume and normalizing the values on a scale from 0 to 100. We collected time-series data for the term “strep throat” for *S. pyogenes* for both the Indianapolis metropolitan area (Google Trends combines Bloomington with the Indianapolis area) and the state of Indiana, covering the period from February 2023 to February 2024. We also collected similar time-series data for the terms “RSV,” “Respiratory syncytial virus,” “COVID,” “COVID-19,” “flu,” and “Influenza” for both the same regions and time periods. This data was aligned with wastewater sample collection dates and weekly search term data for each pathogen. To facilitate matching, search term values were extended to cover all dates within the corresponding week. Pearson correlations and cross-correlations were computed between wastewater concentrations of pathogens against Indianapolis and Indiana-wide search term trends using GraphPad Prism.

## 3. Results and Discussion

### 3.1. Comparison Between crAssphage and PMMoV

**Figure 1:**
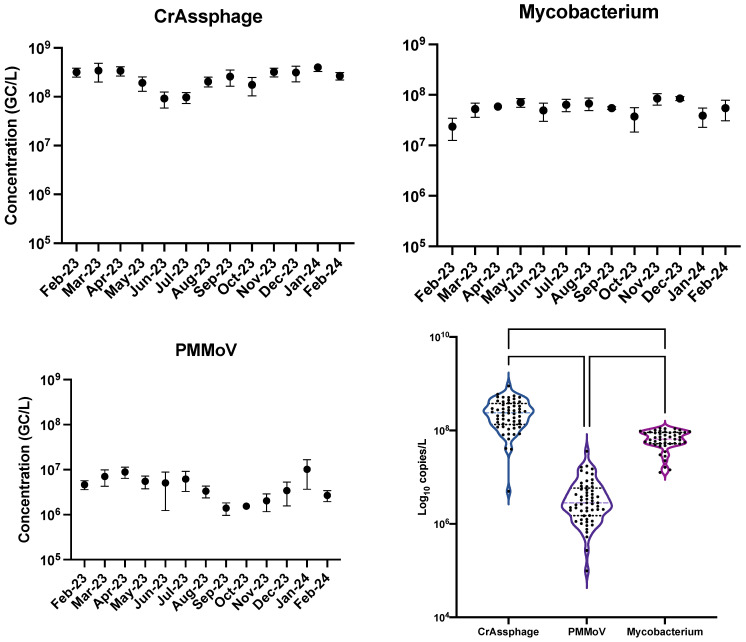
Monthly average concentrations of PMMoV, mycobacterium, and crAssphage in Bloomington wastewater over a year. The bars are the standard deviation for four samples collected each month (*n* = 4). Violin plots on the right show variation in concentrations between crAssphage (*n* = 64), mycobacterium (*n* = 64), and PMMoV (*n* = 64). Asterisks indicate a significant difference between datasets (*p* < 0.0001).

The average monthly concentrations of crAssphage, mycobacterium, and PMMoV over the span of a year in Bloomington, Indiana, were tested for their indication ability and the results are displayed in Figure 1. For each pathogen tested, there remained a steady, high average concentration throughout the time period. The month with the lowest average value of crAssphage detection was October 2023, and the month with the highest average value was August 2023. PMMoV had a different and more variable temporal trend, where August 2023 was the month with the lowest average concentration of PMMoV and July 2023 was the month with the highest concentration. Mycobacterium had the highest average concentration in December 2023 and the lowest average detection in February 2023. The total average concentration for crAssphage, mycobacterium, and PMMoV was 7.26, 7.96, and 5.60 log_10_ gene copies/L, respectively. Mycobacterium was detected consistently throughout the year at statistically similar levels (*p* < 0.05), with the peak concentration being 8.5 log_10_ gene copies/L in December 2023 and the lowest concentration being 5.33 log_10_ gene copies/L in February 2023.

The violin plots in Figure 1 show a trend comparison between the three pathogens. Mycobacterium had a larger spread than both crAssphage and PMMoV, as well as a higher peak concentration. The difference between all three were significant (*p* < 0.0001) with crAssphage having statistically higher concentrations than mycobacterium and PMMoV, and mycobacterium having statistically higher concentrations than PMMoV. Higher concentrations and lower variability over time reveal mycobacterium and crAssphage potential as an improved population normalizer and indicator in wastewater over PMMoV. Reliable population normalizers and fecal indicators require abundant concentrations in wastewater to allow improved sensitivity [27]. Additionally, stability in detection over time is essential for long-term wastewater monitoring for specific pathogens. Our results also agree with several previous studies that show crAssphage abundant concentrations and stability over time in wastewater, making it a more effective tool for wastewater surveillance of pathogens [27,28,29]. Our results are also the first to highlight mycobacterium as a potential population tracker for respiratory bacteria due to its abundance and similarities in shedding with respiratory pathogens, but further evidence is needed to support its continued use. A good wastewater indicator and population normalizer is that they are easily detectable over time, present whenever enteric pathogens are present, survive longer than the strongest enteric pathogens, does not grow in water, and is present in feces [28]. Future studies into mycobacterium’s specific concentration in feces as well as its decay in water will help provide insight into its use as an indicator and normalizer. Alongside previous knowledge of their environmental persistence and resistance to disinfectants, in our study it was proven that mycobacterium remained consistent over time and were independent from disease prevalence [30,31]. There have been a few studies that showed non-tuberculosis mycobacterium presence in wastewater, but none have researched into the possibility of it being used as a population normalizer [30,31].

### 3.2. Temporal Detection of Streptococcus Pyogenes in Wastewater

**Figure 2:**
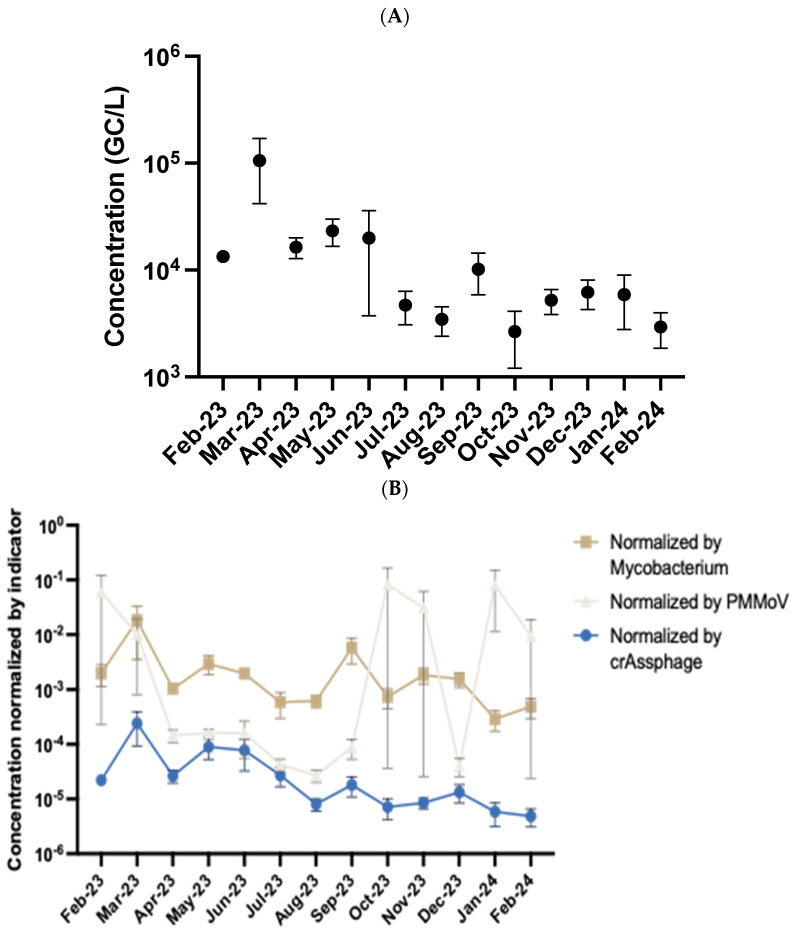
Monthly average concentration of *Streptococcus pyogenes* in Bloomington wastewater over a year. The bars are standard deviation for four samples collected each month (*n* = 4). (**A**) displays the actual concentration of *S. pyogenes* over the year and (**B**) shows the concentration of *S. pyogenes* after normalization of the three indicators.

Throughout the year-long sampling period, *Streptococcus pyogenes* was consistently detected in the majority of wastewater samples, with a positive detection rate of 87%. The average concentration across all samples was 4.25 Log_10_ gene copies per liter. Concentrations peaked during the spring months of 2023 (March–May), reaching a maximum of 5.53 Log_10_ gene copies/L in March. In contrast, the lowest concentrations were observed during the winter months of December 2023 and January 2024, with a minimum value of 3.24 Log_10_ gene copies/L recorded in December. These seasonal trends and overall detection patterns are illustrated in Figure 2A. The concentrations of *S. pyogenes* were then normalized against each of the three indicators tested. Mycobacterium and crAssphage were better normalizers because of their consistent concentrations over time and correlation analysis between concentrations normalized by mycobacterium compared to crAssphage were statistically well correlated (*p* = 0.91) with search term data. Correlation analysis between PMMoV normalized data did not correlate well with search term data (r = 0.10). The normalization of *S. pyogenes* through seasonal trends is displayed in Figure 2B.

WBE has proven to be an effective method for monitoring disease trends, particularly as they fluctuate across different seasons [32,33]. By identifying seasonal patterns in pathogen prevalence, public health agencies can strategically target specific times of the year for intensified mitigation and prevention efforts [32,33,34]. Our findings revealed a clear seasonal trend in the detection of *S. pyogenes*, with significantly higher concentrations observed during the spring months compared to fall, winter, and summer [*p* < 0.05]. Notably, the warmest months, July and August, showed very low levels of *S. pyogenes* in wastewater samples. These observations suggest a potential link between colder weather and the increased prevalence of *S. pyogenes* and related illnesses such as strep throat. This pattern is consistent with prior studies showing that respiratory infections, including those caused by *S. pyogenes,* tend to surge during colder months [35,36]. Several mechanisms likely drive this season increase such as winter-associated reductions in mucosal immunity may impair the body’s ability to fend off pathogens. At the same time, people spend more time indoors in tightly packed, poorly ventilated spaces, conditions that are ideal for facilitating airborne and droplet transmission [37,38]. Moreover, colder weather often brings subtle but important lifestyle shifts, such as decreased physical activity, lower fluid intake, and reduced consumption of fresh produce, all of which can collectively compromise immune resilience [35,36,37,38]. These converging factors can create an environment in which respiratory pathogens can spread more efficiently and infect more hosts. Concentrations of *S. pyogenes* in wastewater were notably low during the cold months of December and January. However, this trend may not reflect an actual absence of the pathogen in the population, but rather a delay in its detectable presence in wastewater. This lag could be attributed to several factors, including the incubation period of infections, lower initial transmission rates, or a delayed onset of symptomatic cases that drive bacterial shedding into the wastewater system through oral fluids [1,3,39,40]. Multiple respiratory pathogens such as SARS-CoV-2, RSV, *Streptococcus*, and *Legionella* have been found to shed in oral fluids, and this therefore shows that WBE is an effective tool for *S. pyogenes* and other types of upper respiratory tract pathogen monitoring in a community. These observations highlight the importance of understanding the temporal dynamics between clinical cases and wastewater signals, especially during seasonal transitions when transmission patterns may shift subtly but significantly.

Specifically, in our community, *S. pyogenes* concentrations were higher in Spring than all other seasons, which directly contrasted with the study done by Shrestha et al. that showed higher pathogen concentrations in winter than in all other seasons. While our study confirms the seasonal presence of *S. pyogenes* in terms of colder and warmer months in the region studied, it is important to recognize that seasonal dynamics may vary based on geography, climate, and cultural behaviors between our region and the region in the previous study [38,41,42]. The concentrations of *S. pyogenes* in our study were also slightly higher than the concentrations observed in the study done by Shrestha et al. These differences could be due to the same differences in regional variations, population sizes, or differences in sample processing and quantification methods. Regional variations such as differences in climate, healthcare access, or rates of community infection, can influence how and when *S. pyogenes* circulates in a population. Population size can result in more wastewater production, which can dilute pathogen concentrations, or conversely, may harbor more cases that contribute to higher detectable levels. Additionally, differences in sample processing and quantification methods, including the timing of sample collection, the concentration and extraction protocols used, and the sensitivity of PCR assays, can all impact the accuracy and comparability of results across studies. As such, future research should integrate wastewater surveillance data with insights into local human behavior and environmental conditions to more comprehensively understand the underlying causes of seasonal trends in disease transmission.

### 3.3. Particle Association of Microorganisms in Wastewater

**Figure 3:**
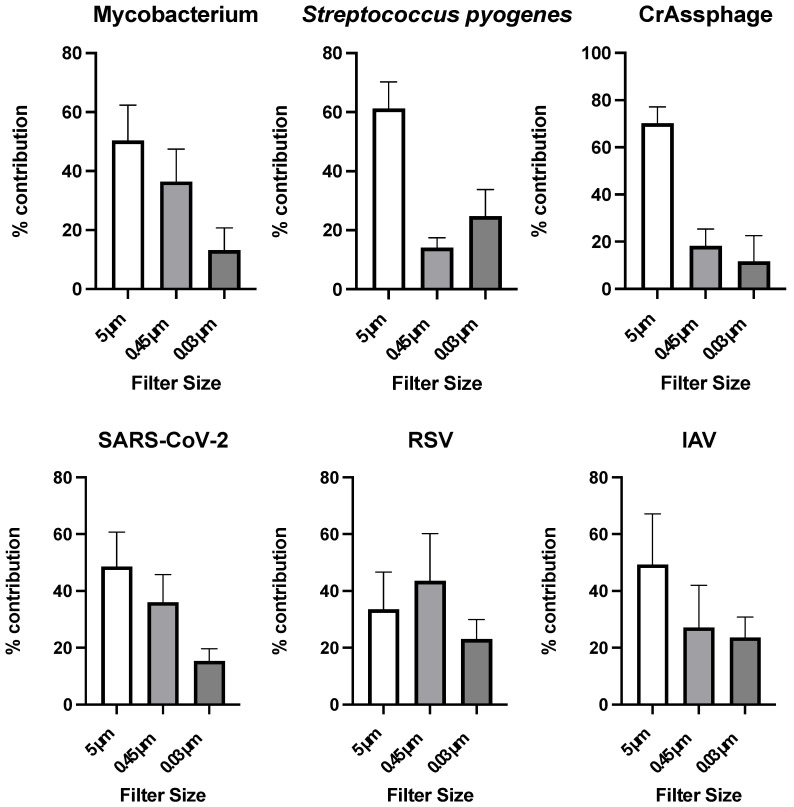
Association of RSV, SARS-CoV-2, IAV, Mycobacterium, *Streptococcus pyogenes*, and crAssphage with particles above 5 um, 0.45 um to 5 um, and below 0.45 um in 5 replicate samples. % contribution on the y axis represents the concentration of microbe on the filter divided by the sum of concentrations for that microbe on all filters.

The association of positive viral and bacterial detections with particles based on filter size contribution is displayed in Figure 3. All of the respiratory pathogens, except for RSV, and crAssphage had the highest concentrations with particles of 5 µm or greater. Mycobacterium, crAssphage, SARS-CoV-2, and IAV all then had the second most contribution from the 0.45 µm filter, and then lastly with the 0.03 µm filter. *Streptococcus pyogenes* had its second highest contribution from the 0.03 µm filter, and then the 0.45 µm filter. CrAssphage had the highest overall percent contribution from the 5 µm filter, with an average contribution of 70.2%. RSV received the highest concentration from the 0.45 µm filter with a percentage of 43.5% contribution, followed by 33.5% from the 5 µm filter, and lastly, the 0.03 µm filter averaged 22.9% contribution.

Our study showed that all targets had concentrations on the 0.03 µm filter size, which has implications for sample processing in wastewater surveillance. Specifically for the bacterial targets, these results show that the molecular assays could also be detecting DNA from dead bacteria, as intact bacteria would not be able to pass through the 0.45 filter (due to the size being larger than 0.45) [43,44]. These results also show that between 80% and 90% of detectable targets are being captured through the use of 0.45 μm filters, which is the common filter used during microbial concentration. This shows that concentration efficiency is high for most targets. All microbial targets had statistically different particle association profiles (*p* < 0.05). These differences could be due to the various outer-layer structural components between the various bacterial pathogens [43,44,45,46]. Pathogens greater than or attached to particles greater than 0.45 µm contributed to most of the detections, resulting in the important of testing the solid portions of wastewater for each target of interest. This size filter did not capture all of the detectable targets though, meaning that there were most likely free-floating pathogens in the liquid portion of wastewater, resulting in this being an important testing media as well [19,47,48,49].

### 3.4. Comparison of Pathogen Wastewater Levels to Publicly Available Data

**Figure 4:**
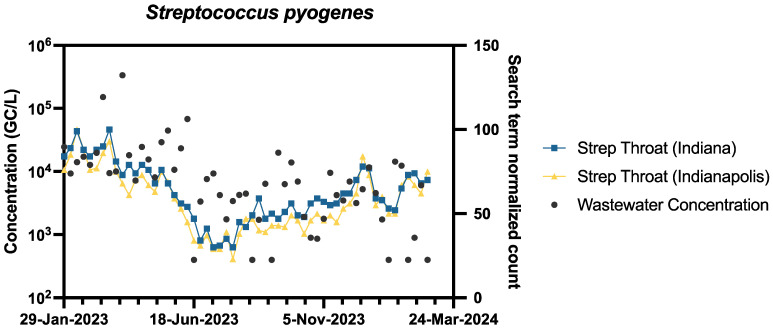
Correlation between wastewater concentrations and search terms for each pathogen. *Streptococcus pyogenes* concentration with “Strep throat” search results in Indiana and Indianapolis with rho values from Spearman’s correlation being r-0.61 and r = 0.58, respectively.

The specific concentration of *S. pyogenes* in wastewater and the search term count of ‘Strep Throat’ for both the state of Indiana and Indianapolis, Indiana specifically were compared and are shown below in Figure 4. When comparing the correlation between the search term and wastewater concentration for *S. pyogenes*, there was a higher correlation of the search term count that covered all of Indiana, 0.61. compared to the count that only covered Indianapolis, Indiana, 0.58. The greatest correlation between the search term and wastewater concentration appears to be in the months of January to February of 2023, and with the lowest correlation being in the summer months, between June to October 2023. The pathogen with the next highest correlation between search terms and wastewater concentration was Influenza A/B. When using the search term ‘Influenza’, 0.50 for Indiana and 0.51 for Indianapolis, there was a higher correlation compared to using the search term ‘Flu’, 0.37 for Indiana and 0.38 for Indianapolis. SARS-CoV 19 showed the same correlation in the Indiana range for both search terms ‘COVID-19’ and ‘COVID’ with a value of 0.36. The correlation differed when narrowing the search term counts to Indianapolis where the term ‘COVID-19’ had the lowest correlation of all pathogens and search terms with a value of 0.25 compared to the search term ‘COVID’ having 0.33. RSV wastewater concentration and search terms, ‘RSV’ and ‘Respiratory syncytial virus’, had correlations of 0.40 and 0.45, respectively, in Indiana and 0.41 and 0.45, respectively, in Indianapolis. Specific correlations for each term are placed in the Supporting Information.

Our results underscore the potential of online search volume data as a valuable supplementary tool for disease surveillance, as we observed moderate correlations between search trends and pathogen levels for the majority of the organisms analyzed in this study, especially *S. pyogenes*. This suggests that monitoring public search behavior can provide real-time insights into disease activity and public awareness. However, there are notable limitations to consider when interpreting this type of data. One key challenge is the influence of media coverage, which can cause sudden spikes in search volume that do not necessarily reflect actual changes in disease incidence [50,51]. Additionally, search volume data generally lacks fine-scale geographic resolution, making it less useful for localized surveillance or community-level interventions [51,52]. As such, while this approach can enhance disease tracking, it should not be used in isolation. To improve accuracy and reduce potential biases, search trend analysis should be integrated with more direct epidemiological data sources, such as clinical reports, laboratory testing (when tested), or wastewater surveillance [12,53]. Combining multiple data streams provides a more comprehensive and reliable picture of disease dynamics and can strengthen early warning systems for emerging public health threats [32,52,53].

### 3.5. Comparison with Other Pathogens Commonly Tested in Wastewater

**Figure 5:**
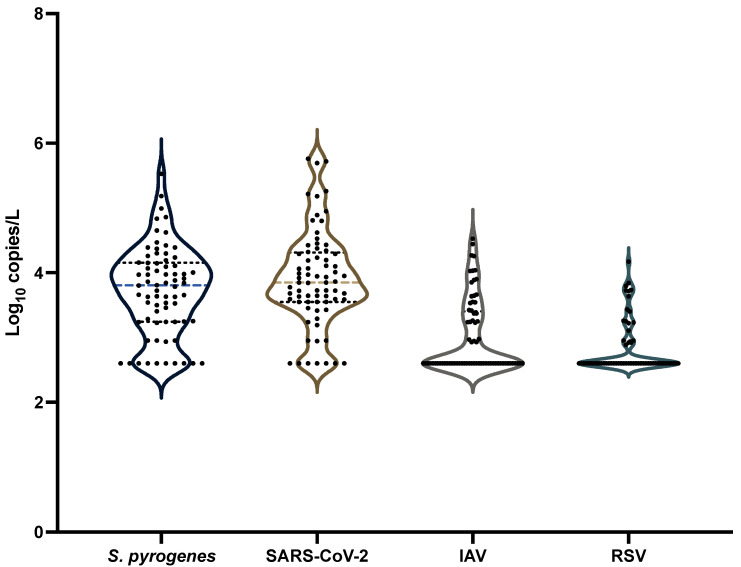
Violin plots showing comparison between *Streptococcus pyogenes* and the various respiratory pathogen concentrations in wastewater over the same period. Samples with non-detections were assumed to be the detection limit (2.6 log_10_ gene copies/L).

When comparing the wastewater concentrations of respiratory pathogens, *S. pyogenes* and SARS-CoV-2 exhibited the highest average levels and the widest distribution patterns. *S. pyogenes* had an average concentration of 4.25 log_10_ gene copies/L, closely aligning with SARS-CoV-2, which averaged 4.60 log_10_ gene copies/L. In contrast, Influenza A virus (IAV) and respiratory syncytial virus (RSV) were detected at lower concentrations, with averages of 3.43 and 3.0 log_10_ gene copies/L, respectively. Among the four pathogens, SARS-CoV-2 showed the highest frequency of detection and the greatest variability in concentration, while RSV was the least frequently detected and had the lowest overall concentrations. These comparative trends in respiratory pathogen prevalence are visualized in Figure 5.

Correlation analysis was performed between all pathogens and there was only a weak correlation between IAV and RSV (r = 0.337). These results suggest that temporal trends between respiratory pathogens do not correlate, and that each individual respiratory pathogen, including *S. pyogenes*, follows its own trend within this calendar year. Prior studies carried out with viruses that depend on each other (adenovirus and adeno-associated virus) have shown similar trends between viruses, but as none of the current pathogens require any of the others for infection, temporal trends are significantly different [13,54,55]. Temporal trends between pathogens could also be significantly different due to specific mechanisms of transmission between each pathogen and the particular vulnerable groups that are affected by them [42,56]. As pathogen trends result from the various complex relationships in human activities and microbial dynamics, it is difficult to connect trends between various respiratory pathogens [41,55]. Prior studies which have measured trends in wastewater respiratory pathogens have also observed similar differences between the temporal trends of various pathogens [54,55]. This suggests that *S. pyogenes* temporal trends are unique when compared to other common respiratory pathogens that are circulating within the same community.

### 3.6. Limitations

One of the primary limitations of this study is the absence of corresponding clinical case data, largely due to limited disease surveillance in the region. Without access to reliable clinical case counts, we were unable to directly compare the concentration of viral markers in wastewater to actual infection rates within the community [57]. This gap hinders our ability to determine how accurately wastewater signals reflect ongoing public health trends. To bridge this gap, future research should incorporate clinical surveillance data alongside wastewater analysis to better evaluate whether the presence of pathogens in wastewater correlates with active infections among the population.

## 4. Conclusions

In conclusion, this study makes an important contribution through the detection and quantification of *S. pyogenes* over time and compares it to other wastewater targets. The findings revealed that *S. pyogenes* is highest in the spring and significantly associates with larger particles in wastewater. The results also indicate that *S. pyogenes* does not follow the same infection patterns as other endemic respiratory viruses in the community, which might suggest the coinfection of certain pathogens could be a rare occurrence. Overall, this research highlights the potential of WBE to provide important insights into respiratory health trends to improve public health mitigation strategies within smaller communities.

## Data Availability

The original contributions presented in this study are included in the article/Supplementary Materials. Further inquiries can be directed to the corresponding author(s).

## Supplementary Materials

The following supporting information can be downloaded at: https://www.mdpi.com/article/10.3390/pathogens14070658/s1, Figure S1: Strep Throat search data correlation with normalized wastewater concentrations and actual concentration.; Figure S2: Correlation between wastewater population normalizers; Figure S3: Correlation of S. pyrogenes to other commonly tested respiratory pathogens. These pathogens were tested in the same wastewater samples over the same sampling period.; Table S1: Primers used in this study; Table S2: Google Trend correlation with wastewater data.
