# Supplementary material for: Wastewater Surveillance for Group A Streptococcus pyogenes in a Small City
Source: Pathogens. 2025 Jul 3;14(7):658. doi: 10.3390/pathogens14070658 (PMC12298068; doi:10.3390/pathogens14070658)
Supplement: Supplementary file 1 [file pathogens-14-00658-s001.zip › pathogens-3635763-supplementary.pdf]

Supporting Information: Wastewater Surveillance for Group A Streptococcus pyogenes in a small City

Olivia Birch[a], Frankie Garza[a], and Justin Greaves[a]

[a]Department of Environmental and Occupational Health, School of Public Health

Indiana University-Bloomington

Corresponding Author:

Justin C.J. Greaves

jcgreave@iu.edu

Indiana University-School of Public Health

2719 E 10th Street, Innovation Center

Bloomington Indiana, 47408

Keywords

Wastewater based epidemiology, GAS pharyngitis (strep-throat), Viral respiratory pathogens, Filtration, Bacteria

Methods:

Table S1. Primers used in this study.

Organism	PPM	Name	Sequence	Source
SARS-CoV-2	Forward	2019-nCoV_N1-F	GACCCCAAAATCAGCGAAAT	Sherchan et al. [20]
	Reverse	2019-nCoV_N1-R	TCTGGTTACTGCCAGTTGAATCTG	
	Probe	2019-nCoV_N1-P	FAM-ACC CCG CAT TAC GTT TGG TGG ACC- 3BHQ_1	
InFA	Forward	InfA For-1	CAA GAC CAA TCY TGT CAC CTC TGA C	Boehm et al. [21]
	Reverse	InfA Rev-1	GCA TTY TGG ACA AAV CGT CTA CG	
	Probe	InfA Probe	FAM - TGC AGT CCT /ZEN/ CGC TCA CTG GGC ACG - 3BHQ_1	
RSV	Forward	RSVAB F	CTCCAGAATAYAGGCATGAYTCTCC	Hughes et al. [22]
	Reverse	RSVAB R	GCYCTYCTAATYACWGCTGTAAGAC	
	Probe	RSVAB P	HEX - TAACCAAATTAGCAGCAGGAGATAGATCAG - 3BHQ_1	
PMMoV	Forward	PMMV-FP1	GAGTGGTTTGACCTTAACGTTTGA	Kitajima et al. [23]
	Reverse	PMMV-FP1-rev	TTGTCGGTTGCAATGCAAGT	
	Probe	PMMV-Probe1	FAM-CCTACCGAAGCAAATG-NFQ-MGB	
CrAssphage	Forward	CPQF	CAG AAG TAC AAA CTC CTA AAA AAC GTA GAG	Greaves et al. [16]
	Reverse	CPQR	GAT GAC CAA TAA ACA AGC CAT TAG C	
	Probe	CPQ56P	FAM- AAT AAC GAT TTA CGT GAT GTA AC-BHQ-1	
Streptococcus pyogenes	Forward	spy1258F	GCA CTC GCT ACT ATT TCT TAC CTC AA	Kodani et al. [24]
	Reverse	spy1258R	GTC ACA ATG TCT TGG AAA CCA GTA AT	
	Probe	spy1258P	FAM - CCG CAA CTC ATC AAG GAT TTC TGT TAC CA - 3BHQ_1	
Pan mycobacterium	Forward	110F	CCTGGGAAACTGGGTCTAAT	Aoki et al. [25]
	Reverse	I571R	CGCACGCTCACAGTTA	
	Probe	H19Rm	HEX – TTTCACGAACAACGCGACAAAC - 3BHQ_1	

Statistical Analysis:

Correlation statistics were done in GraphPad Prism assuming normal distribution and using Pearson's r correlation that provided a number between -1 and 1. All correlation statistics were done using the wastewater sampling days which were the exact same days for which the search data was also obtained (weekly value between February 1st, 2023 and February 24th, 2024.

Results:

Table S2. Google Trend correlation with wastewater data.

**Search Term**	**Indiana**	**Indianapolis**
Strep Throat	0.61	0.58
Flu	0.37	0.38
Influenza	0.50	0.51
Covid-19	0.36	0.25
Covid	0.36	0.33
RSV	0.40	0.41
Respiratory syncytial virus	0.45	0.45

Figure S1: Strep Throat search data correlation with normalized wastewater concentrations and actual concentration.

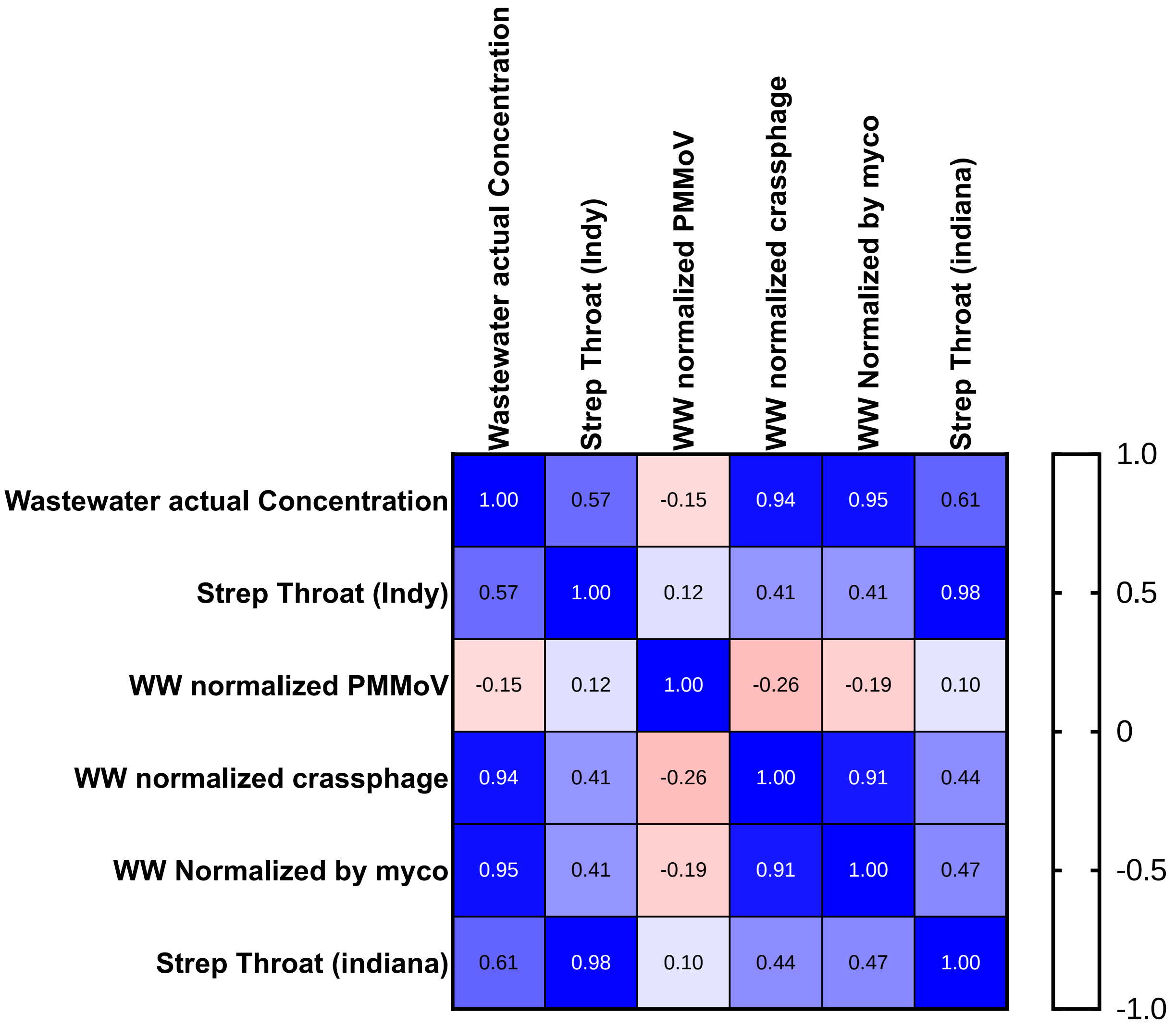

Figure S2: Correlation between wastewater population normalizers.

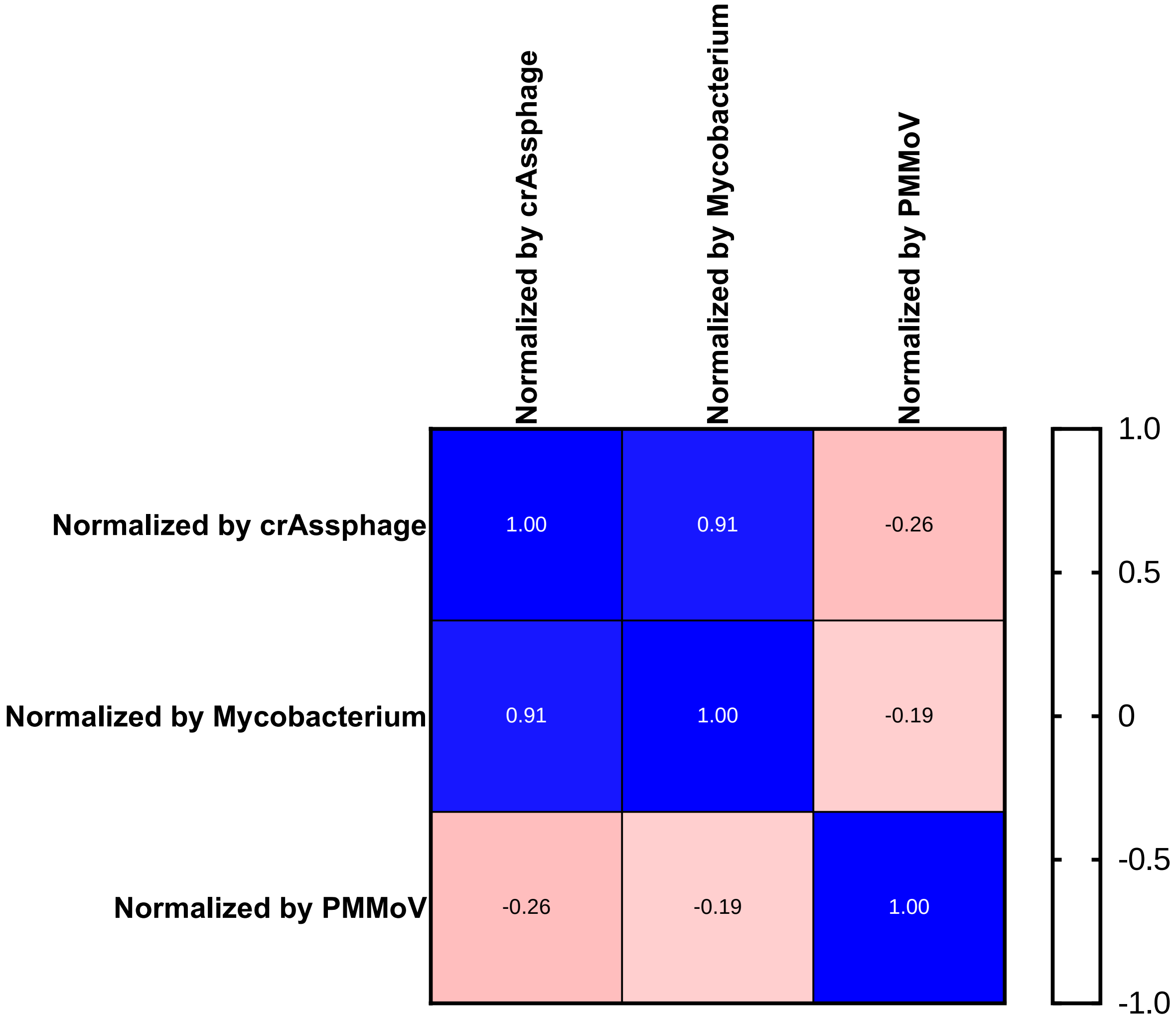

Figure 3: Correlation of *S. pyrogenes* to other commonly tested respiratory pathogens. These pathogens were tested in the same wastewater samples over the same sampling period.

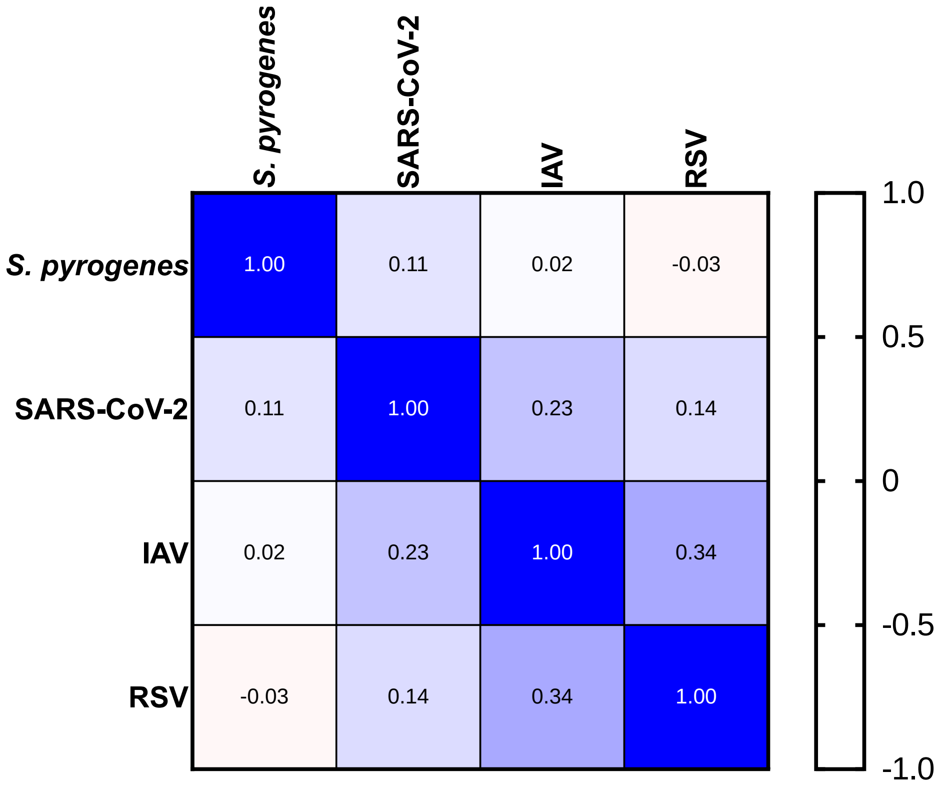

REFERENCES

1. Sherchan, S.P., et al., *First detection of SARS-CoV-2 RNA in wastewater in North America: A study in Louisiana, USA.* Science of The Total Environment, 2020. **743**: p. 140621.
2. Boehm, A.B., et al., *Wastewater concentrations of human influenza, metapneumovirus, parainfluenza, respiratory syncytial virus, rhinovirus, and seasonal coronavirus nucleic-acids during the COVID-19 pandemic: a surveillance study.* Lancet Microbe, 2023. **4**(5): p. E340-E348.
3. Hughes, B., et al., *Respiratory Syncytial Virus (RSV) RNA in Wastewater Settled Solids Reflects RSV Clinical Positivity Rates.* Environmental Science & Technology Letters, 2022. **9**(2): p. 173-178.
4. Kitajima, M., H.P. Sassi, and J.R. Torrey, *Pepper mild mottle virus as a water quality indicator.* npj Clean Water, 2018. **1**(1): p. 19.
5. Greaves, J., et al., *Persistence of emerging viral fecal indicators in large-scale freshwater mesocosms.* Water Research X, 2020. **9**.
6. Kodani, M., et al., *Application of TaqMan Low-Density Arrays for Simultaneous Detection of Multiple Respiratory Pathogens.* Journal of Clinical Microbiology, 2011. **49**(6): p. 2175-2182.
7. Aoki, M., et al., *Quantitative detection and reduction of potentially pathogenic bacterial groups of Aeromonas, Arcobacter, Klebsiella pneumoniae species complex, and Mycobacterium in wastewater treatment facilities.* Plos One, 2023. **18**(9).
